# Improving Patient Access to Hospital Pharmacists Using eConsults: Retrospective Descriptive Study

**DOI:** 10.2196/38518

**Published:** 2023-01-27

**Authors:** Vera Weinberg, Eva van Haren, Kim B Gombert-Handoko

**Affiliations:** 1 Leiden University Medical Center Leiden Netherlands

**Keywords:** eConsult, online consultation, eHealth, electronic patient portal, hospital pharmacy, medication reconciliation

## Abstract

**Background:**

eConsults are increasingly used worldwide to reduce specialist referrals and increase access to medical care. An additional benefit of using an eConsult tool is a reduction of health care costs while improving the quality of health care and patient participation. Currently, shared decision making is increasingly implemented and preferred by patients. eConsults are also a promising tool to improve access to the hospital pharmacist. Patients often have questions about their medication. When medication is started during a hospital admission or outpatient visit, community pharmacists are not always sufficiently informed to answer patient questions. Direct contact with hospital pharmacists may be more appropriate and efficient. This contact is facilitated through the eConsult feature in the hospital’s patient portal.

**Objective:**

This study aims to evaluate the prevalence and contents of the eConsults sent by patients to hospital pharmacists.

**Methods:**

A first retrospective descriptive study was conducted at the Leiden University Medical Center in the Netherlands. Patients who sent at least one eConsult to a hospital pharmacist between March 2017 and December 2021 were included. Patient characteristics and the number of medications taken were extracted from electronic health records. The content of eConsults was analyzed and grouped into different subjects. Time of sending of the eConsults was analyzed. A comparison was made between the number of eConsults sent to the hospital pharmacy and the number sent to the medical center. Finally, the appropriateness for evaluation by the hospital pharmacist was assessed in all eConsults.

**Results:**

During the study period, 983 eConsults (from 808 patients) were sent to the hospital pharmacist. The average patient age was 56 (SD 15.9) years, and 51.4% (415/808) were male; 47.8% (386/808) of the patients used 0 to 4 medications, 33.0% (267/808) used 5 to 9 medications, and 19.2% (155/808) used ≥10 medications. Of the eConsults, 10.9% (107/983) were excluded due to not being medication-related or not intended for the hospital pharmacist. Patients being treated in 31 medical specialties sent eConsults to the hospital pharmacist. The most common medical specialty was cardiology with 22.5% (197/876) of the eConsults. Most eConsults were sent during office hours (614/876, 70.2%). eConsult subjects were medication verification (372/876, 42.5%), logistics (243/876, 27.7%), therapeutic effect and adverse events (100/876, 11.4%), use of medication (87/876, 9.9%), and other subjects (74/876, 8.4%).

**Conclusions:**

Introducing eConsults allows patients to ask medication-related questions directly to hospital pharmacists. Our study shows that patients send medication reconciliation–related eConsults most often. Use of the eConsult tool leads to fast, direct, and documented communication between patient and hospital pharmacist. This can reduce medication-related errors, improve patient empowerment, and increase access to the hospital pharmacist.

## Introduction

As a result of increased access to the internet, eHealth technology is rapidly developing; health services offered include electronic health records (EHRs) [1,2]. Currently, most hospitals in the Netherlands have integrated EHRs and created patient portals for their patients. In 2021, the Dutch National Institute for Public Health and the Environment reported that 75% of medical specialists consult with patients remotely through an EHR [3]. Using the patient portal, patients can view test results and make appointments with their health care specialists [4]. Furthermore, patients have the opportunity to exchange information and communicate securely with their health care providers through electronic consultations by chat (eConsults).

In an environmental scan published by Joschko et al [5], implementation of eConsults was found to have increased worldwide. The aim of eConsults is to complement and facilitate usual care and not to replace it. This is in line with the World Health Organization recommendations on digital interventions for health system strengthening [6]. When face-to-face contact is not possible (eg, during COVID-19 pandemic lockdowns) or necessary, use of patient portals facilitates access. eConsults were a convenient solution to provide care without risk of spreading the virus and therefore increasingly used during the pandemic [7]. Besides facilitating access, use of eConsults improves the quality of health care by increasing patient participation and shared decision-making. Unnecessary specialist visits are avoided and medical costs are reduced [2,5,8,9]. In a 2015 systematic review by Vimalananda et al [10], 27 studies on eConsult services were reviewed. The authors found high levels of provider satisfaction (70%-95%), faster replies (less than 3 days), and a decline in specialist referrals. Barriers to implementing an eConsult tool include low levels of patient engagement and awareness, staff inexperience and limited availability, and lack of suitability for all patient groups and financial obstacles [7].

Because of the benefits of an eConsult tool for patient care, the hospital pharmacy of the Leiden University Medical Center (LUMC) integrated a medication-related eConsult tool; the first hospital in the Netherlands to do so. Using the eConsult tool, patients could consult their hospital pharmacist electronically by entering their question or comment in their EHR linked to a specific prescribed drug. Advantages of an eConsult over a video consult, email, or chat are that health care specialists and patients can send and reply to the consult at their preferred time and eConsults can be linked to patient-specific information in the EHR. A chatbot could also be implemented for such purposes; however, in our case patient-specific answers are often needed and real-life contact with a specialist is preferable.

Hospital pharmacists do not have regular consultation hours and are not always visible and approachable for patients. Using the eConsult tool, patients can access an overview of their medications and ask a hospital pharmacist questions about their medication. Whether a hospital pharmacist eConsult feature would be a useful addition to the patient portal has not yet been determined, but the eConsult tool may be a promising way to facilitate access to the hospital pharmacist. Furthermore, with the use of eConsults, patients can play an important role in the management of their own health care. Research demonstrates that patient-mediated medication reconciliation through eHealth can improve medication safety. Heyworth et al [11] found that over two-thirds of 60 enrolled patients had at least one discrepancy in their medication overview. In addition, almost one-third of enrolled patients had at least one potential adverse drug event due to a discrepancy in their medication overview. Patients participated with enthusiasm and contributed to the correct use and documentation of their own medication [12].

The aim of this study was to create an overview of the patient population that uses pharmacist-patient eConsults with respect to their medication and subjects of their eConsults. This is the first descriptive study of the use of eConsults in the hospital pharmacy and a first exploration of the eConsult tool. Consequently, we will improve our insight into the needs of our patients regarding pharmaceutical care, optimize the eConsult tool, and share our findings with other hospital pharmacies.

## Methods

### Data Extraction and Categorization

A first descriptive study was performed at the LUMC, a Dutch academic hospital with approximately 880 hospital beds (in 2018) that provides mostly specialized care. Patients who sent at least one eConsult to a hospital pharmacist between March 2017 and December 2021 were included in this study. eConsults from patients were answered on a daily basis by a resident hospital pharmacist during office hours.

eConsults were extracted from the EHR and entered into an Excel (2017, Microsoft Corp) spreadsheet. The patient characteristics age, gender, and number of medications used were collected from the EHR. The content of the eConsults were reviewed independently by two researchers. To ensure similarity in reviewing the content, the first 100 eConsults reviewed were validated by the other researcher.

eConsults were analyzed using descriptive statistics and categorized into subjects and medical specialties by two authors working independently. The following subjects were used to categorize the eConsults: medication verification, logistics, therapeutic effect and adverse events, use of medication, and other subjects. These subjects were determined by the hospital pharmacist based on experience answering eConsults during daily practice. *Other subjects* was chosen to ensure that all eConsults could be categorized.

The number of eConsults and times sent were investigated. To compare the pharmacist eConsults with the number of eConsults sent by patients to the hospital, eConsult data from the entire hospital were extracted from the EHR and analyzed. Finally, the relevancy of the eConsults for the hospital pharmacist was assessed. Relevancy for the hospital pharmacist was defined as medication-related eConsults. The spreadsheet was populated and analyzed using descriptive statistics in Excel. We encourage data availability, and access to our data can be requested from the corresponding author.

### Ethics Approval

The Medical Research Involving Human Subjects Act (WMO, in Dutch) was not applicable because the study was observational, required no participant involvement, and used only LUMC data. For this reason, written informed consent from the patient was not required for participation in this study in accordance with the national legislation and the institutional requirements [13]. The study data were anonymized before analysis. The eConsults were stored in the private patient portal, and patient privacy and confidentiality were ensured. No compensation was given to the participants who sent eConsults.

## Results

### Development and Implementation Phase of the eConsult Tool

In 2014, the eConsult tool was developed at the LUMC for the renal diseases and medical oncology departments. The tool was developed for patient-physician interaction. In the years after, the tool was implemented in several other departments, including the hospital pharmacy in 2017. First, a medication section to be used in the pharmacy patient portal was implemented. This consisted of an overview of the medication registered in the EHR, reference to a patient-friendly drug information site, and option to send an eConsult labeled “Ask the hospital pharmacist” as well as an explanation on why this insight is needed.

In our hospital, we were one of the first medical departments to offer eConsults to patients. Since every patient has a medication record, we decided every patient could consult the hospital pharmacist. Patients’ eConsult questions and pharmacist responses are available in EHRs for other medical specialists involved in the treatment. We expected the burden on medical specialists to decrease when relevant questions were answered by pharmacists.

A tool developed to allow patients to request for repeat prescriptions in the medication overview routed requests to prescribers. The pharmacist received a lot of questions not intended for them, making the tool inefficient. After the tool was optimized, it was more useful and user-friendly for the pharmacists.

### Results of the First Years Using the eConsult Feature

During the 5-year study period, 983 eConsults involving 808 patients were received and analyzed. Of these 808 patients, 120 patients sent multiple eConsults. Patient characteristics are summarized in Table 1.

**Figure 1:**
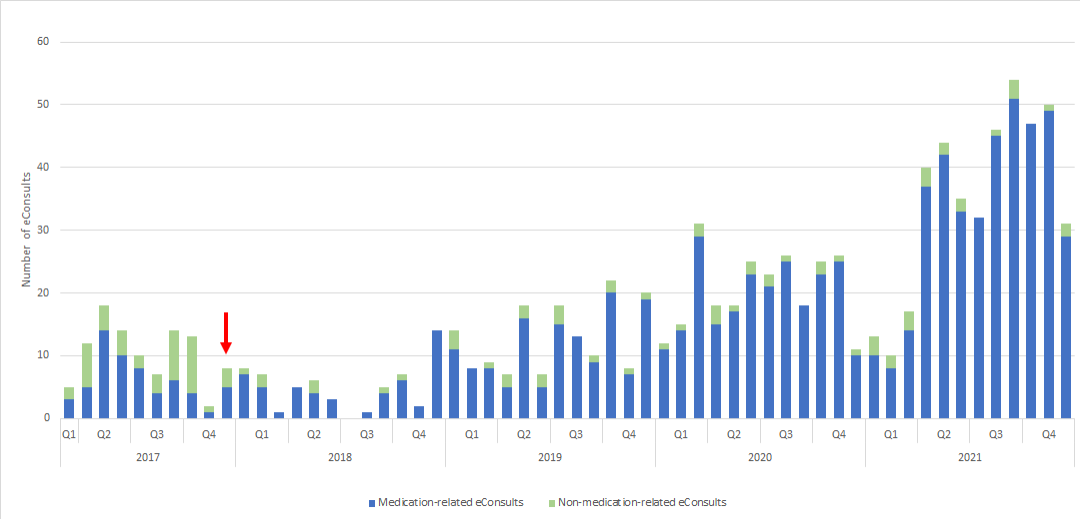
Number of eConsults sent monthly to the hospital pharmacist (2017-2021), with the red arrow indicating the moment the feature was adjusted to reduce the amount of non–medication-related eConsults. Q1 of 2017 only consists of the month March.

**Figure 2:**
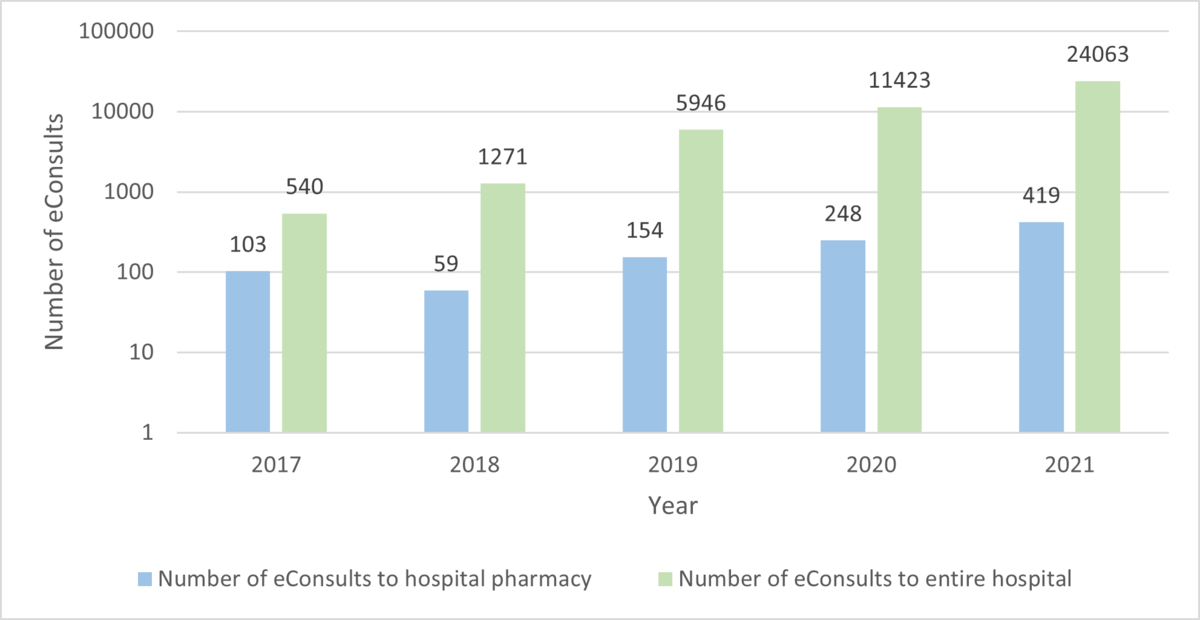
Number of eConsults sent annually to the hospital pharmacist and entire hospital.

Figure 1 shows the number of eConsults sent every month to the hospital pharmacist, in which the distinction between medication-related and non–medication-related eConsults is also shown. In Figure 2, the number of eConsults sent annually to the hospital pharmacy is shown in comparison to the total number of eConsults sent to the hospital. This figure also shows that the number of eConsults sent to the LUMC increased faster than the number sent to the hospital pharmacy of the LUMC.

After data analysis, the 10.9% (107/983) of eConsults that were irrelevant or not intended for the hospital pharmacist were excluded (eg, non–medication-related). The non–medication-related eConsults were, for example, questions about physician appointments, questions about lab/scan results, or disease-related concerns. In addition, as mentioned before many eConsults concerned prescription refills. Most eConsults were sent during office hours (615/876, 70.2%), and medication verification was the most common topic (372/876, 42.5%). Table 2 presents an overview of the subjects included in each category and a representative patient question from that category. Additionally, the number of medication-related eConsults sent to the hospital pharmacist per category is shown in Table 2. Patients being treated in 31 medical specialties sent eConsults to the hospital pharmacist, of which the most common specialty was cardiology, with 22.5% (197/876) of the eConsults. The number of eConsults by other common medical specialties is demonstrated in Table 3.

**Table 1:** Characteristics of users of the hospital pharmacist eConsult tool (n=808).

Characteristic		Value, n (%)
**Sex**		
	Male	415 (51.4)
	Female	393 (48.6)
Age (years), mean (SD)		56 (15.9)
**Number of medications taken**		
	0-4	267 (33.0)
	5-9	117 (14.5)
	10-14	29 (3.6)
	15-19	9 (1.1)
	≥20	267 (33.0)

**Table 2:** Explanation of the categories of eConsults sent to the hospital pharmacist by the patients and the number of medication-related eConsults sent by patients in every category (n=876).

Category	Content of eConsult	Example of patient eConsult	eConsults sent, n (%)
Logistics	Repeat prescriptions Availability of medication Delivery of medication at home	“My medication supply is almost finished. When will my medication be delivered or when can I pick up my medication?”	243 (27.7)
Use of medication	Shelf life of medication Storage conditions of medication Dosing schedules Interval between and order of medications intake	“Last night I forgot to store my chloramphenicol eye drops in the fridge. Can I still use these drops?”	87 (9.9)
Medication verification	Updates on the hospital medication overview Questions about generic and brand names of medication Updates on dose adjustments, discontinuation of medication, and starting new medication	“In the medication overview of my electronic health record, I see the drug venlafaxine. I don’t use that drug anymore; I switched to sertraline 100 mg once a day. Can you adjust this in my medication overview?”	372 (42.5)
Therapeutic effect and adverse events	Mechanism of action of medication Drug-drug interactions or drug-food (supplement) interactions Adverse events Need for co-medication Difference in effect of different medication brands Influence of pharmacogenetics on medication	“I have read about interactions between grapefruit juice and medication. Can I drink grapefruit juice in combination with the medication that I use?”	100 (11.4)
Other	Actual medication overviews for traveling Insurance and reimbursement of medication	“For my trip to the United States, I need an actual medication overview. Can you send this to me?”	74 (8.4)

**Table 3:** Classification of eConsults according to medical specialty of the provider treating the patient (n=876).

Classification		Value, n (%)
**Medical specialty**		
	Cardiology	197 (22.5)
	Neurology	77 (8.8)
	Rheumatology	71 (8.1)
	Gastroenterology	59 (6.7)
	Endocrinology	50 (5.7)
	Oncology	46 (5.3)
	Ophthalmology	46 (5.3)
	Nephrology	38 (4.3)
	Dermatology	32 (3.7)
	Internal medicine	32 (3.7)
	Gynecology	31 (3.5)
	Surgery	27 (3.1)
	Otorhinolaryngology	25 (2.9)
	Hematology	25 (2.9)
	Pulmonary medicine	24 (2.7)
	Transplantation	21 (2.4)
	Urology	14 (1.6)
	Other^a^	61 (7.0)
**Sent during office hours (0900-1700)**		
	Yes	615 (70.2)
	No	261 (29.8)

^a^The medical specialties anesthesiology, clinical genetics, fertility, infectious diseases, neurosurgery, oral and maxillofacial surgery, orthopedics, pain medicine, pediatrics, plastic surgery, psychiatry, rehabilitation, vascular medicine, and unknown specialties had fewer than 10 eConsults each (n=107).

## Discussion

### Principal Findings

This study aimed to evaluate the prevalence and contents of the eConsults sent by patients to hospital pharmacists. The results show that of the 876 eConsults that were medication-related, 42.5% concerned updates on the medication overview, 11.4% concerned questions about therapeutic effect and adverse events, and 9.9% about the use of medication. Additionally, the number of eConsults sent to the hospital pharmacy increased over time. Of the patients who sent at least one eConsult, the average age was 56 (SD 15.9) years and 51.4% were male. The most common medical specialty that the eConsults concerned was cardiology.

The results of this study show that the medication overview in the EHR is often incorrect or incomplete. It is important to obtain a complete and accurate medication overview in the hospital. If this is not the case, patients are at risk of medication errors (ie, receiving the wrong medication, not receiving the required medication), which can lead to adverse events [14]. It might be possible that without the eConsult tool, medication updates are missed and patients get the wrong medication or dose when hospitalized or after transitioning to primary care.

Besides medication verification, eConsults contained questions about therapeutic effect and adverse events (11.4%) and use of medication (9.9%). These requests were very specific and were best answered by a hospital pharmacist. For example, questions concerned CYP-enzyme interactions, pharmacogenetics, storage stability, shelf life of medication, and creating clear dosing schedules for patients. Without the eConsult tool, patients will probably ask these questions to their doctor during a consult. Subsequently, the doctor will pass a lot of these questions to the pharmacist. The introduction of the feature can prevent this inefficient way of communication and save time for doctors, pharmacists, and patients. Moreover, research by Skeith et al [12] demonstrated that the use of eConsults reduced the number of specialist referrals and visits, which potentially results in cost savings to the health care system. Implementation of the tool in the hospital pharmacy could lead to a further decrease in specialist referrals and visits and therefore will indirectly be cost saving. Even though earlier research showed that eConsults will lead to fewer in-person referrals and may lead to reduced quality of these consults, this would not affect pharmacist-patient consults because there is no direct contact between patient and pharmacist in daily practice in the Netherlands [15]. In contrast, by implementing the eConsult tool, the pharmacist-patient contact even increases.

The results of this study show a substantial increase in eConsults sent over time to the hospital pharmacy and to the LUMC in general (Figures 1 and 2). This increase can be partially explained by the rising eHealth literacy among the population. The growth in volume of eConsults sent was not only associated with an increase of unique users but is also partially explained by the reuse of the tool by 120 of the 808 individual patients. Our results align with the results of Tak et al [16], who have shown that participation of patients in decision making concerning their own health care may lead to an increase in resource utilization. Furthermore, they showed that patient participation is important for shared decision making, which has a positive influence on health outcomes and patient satisfaction. Palen et al [17] demonstrated that when patients have online access to their clinicians and patient portal, this may lead to an increase in use of clinical services in comparison with patients who did not have online access. From this it can be suggested that online access to patients’ own health care record may lead to an increase in use of health services due to additional health concerns identified through online access. Our results suggest that increased accessibility to hospital pharmacists (and other medical specialists) triggers patients to ask questions and share their concerns about their health care more frequently, confirming previous assumptions.

Other explanations for the increase of eConsults sent over time could be habituation of using eConsults and integration of the tool by other specialisms [18]. In addition, the COVID-19 pandemic could be a reason for the increase of eConsults sent over time. A poll from the consumer panel of health care of the Netherlands Institute for Health Services Research showed that more than half of the Dutch population started using eHealth more during the COVID-19 pandemic [19]. Remarkably, the increase in the number of eConsults sent to the LUMC in general was higher than sent to the hospital pharmacy. This can be explained by the increase in departments using the eConsult tool. In 2017, the hospital pharmacy was one of the 28 early adopting departments using eConsults for patient-provider communication. Since then, use of the tool has increased to 52 departments in 2021.

During the study period, 808 patients sent at least one eConsult. The average age of patients was 56 (SD 15.9) years, which is relatively high in comparison with other published research. This indicates that the tool is usable for elderly patients. Zanaboni et al [20], Lowenstein et al [21], and Wang et al [18] looked into patient use of online access to EHR, among other things. Results by Zanaboni et al [20] showed that the use of the eConsult service was lower for people aged over 55 years. Wang et al [18] showed an average age of 44 years for patients using eConsults, and Lowenstein et al [21] showed an average age of 48 years. Most patients who sent eConsults to the hospital pharmacist were being treated in the department of cardiology. A possible explanation is that patients treated for cardiac diseases are commonly polypharmacy patients. However, results could be distorted because the department of cardiology has not yet implemented its own eConsult tool, and questions a patient would normally ask their cardiologist were possibly directed to the pharmacist. Hoogenbosch et al [22] looked into the predictive characteristics of patients who use eConsults. Besides chronic illness, eHealth literacy was a significant predictive characteristic.

### Strengths and Limitations

The suitability of the eConsult to provide access for primary health care providers to the hospital pharmacist has previously been established [23]. However, to our knowledge no further research has been published describing the use of patient-pharmacist eConsults in pharmaceutical care. Besides being innovative, the strengths of this study are the extensive study period (5 years) and number of eConsults examined (n=876).

Additionally, this study has several limitations. Primarily, no causality can be established between the parameters studies due to the descriptive and retrospective nature of the study. It would be of added value to explore possible correlations between, for example, the number of prescribed drugs and the number of eConsults sent or between the topic of the eConsults and the medical specialty. However, as this is a first exploration of the feature, limited data are available (eg, on the total number of patients treated by every department and their characteristics). Additionally, the number of prescribed drugs was characterized into groups (Table 1) instead of analyzing the amount as a continuous variable. This makes it impossible to establish a correlation between, for example, the number of prescribed drugs and the number of eConsults per patient.

The third limitation is that changes have been made to the patient portal to optimize the eConsult tool during the study period. At the end of 2017, we performed an interim analysis in which we found that a quarter of the eConsults were not relevant for the hospital pharmacist (eg, requests for doctor appointments or repeat prescriptions and questions concerning lab or test results not related to pharmaceutical care). Answering these irrelevant eConsults is cumbersome and not satisfying for the patient nor the hospital pharmacist. To clarify what type of questions could be asked to the hospital pharmacists, different subjects were added to the eConsult tool in the patient portal during the study period. The result of this successful intervention was that at the end of the study period, only 10% of eConsults were not relevant or not intended for the hospital pharmacist (Figure 1). The reduction of non–medication-related eConsults over the study period is also shown in Figure 1. To ensure the quality of eConsults, questions should be directed to the most suitable health care professional. Therefore, it is now possible to forward the eConsults to these medical specialists. Furthermore, to ensure that the requests for repeat prescriptions were redirected automatically, an additional feature was implemented in the tool.

### Future of the eConsult Tool

Based on this study, we present a few recommendations. The first is to add more subjects to the LUMC’s eConsult tool to further reduce the number of irrelevant eConsults sent. For example, categories integrated in this study can be added to the tool: interactions and side effects of medication, use of medication, updates on the medication overview, storage of medication, and traveling with medication. For further clarification, sample questions could be included in the different categories. Second, an evaluation functionality allowing patients to rate and comment on the eConsult tool should be integrated into the tool. The fact that more eConsults were sent later in the study period suggests that patients appreciate the opportunity to send eConsults (Figure 1). It is important to investigate patient satisfaction and needs with the evaluation functionality. With this information, outcomes as adherence and medication knowledge can be determined for our population and, if needed, improved further. A comparative study between the patient population that uses the eConsult tool and a patient population that does not use the tool would be insightful to determine the effect of the tool measured by outcomes as adherence and medication errors.

With the growth of the tool, it could become cumbersome for the hospital pharmacist to answer all the eConsults. Therefore, future eConsults concerning medication reconciliation could be redirected to pharmacy technicians at the hospital. Furthermore, it could be useful to create subcategories like dose adjustment, discontinuing medication, or adding new prescriptions.

This study shows that the implementation of the eConsult tool can improve the visibility of and access to the hospital pharmacist for the patient. The use of the eConsult tool resulted in an increase in patients involved in their own medication management. In particular, discrepancies in the hospital medication overview were corrected due to the active role of the patient. For these reasons, we would recommend the use of a pharmacy eConsult tool to every hospital pharmacy.

### Conclusions

This study shows that eConsults sent by patients to the hospital pharmacist mainly concerned medication reconciliation. Use of the eConsult tool leads to fast, direct, and documented communication between patient and hospital pharmacist. This can reduce medication-related errors, improve patient empowerment, and increase access to the hospital pharmacist. Although the eConsult tool at LUMC should still be optimized, and patient satisfaction needs to be investigated, we recommend the use of patient-pharmacist eConsults to every hospital pharmacy because it is a useful tool for patients to rapidly obtain nonurgent, patient-specific, expert advice from hospital pharmacists.

## Abbreviations

eConsult: electronic consultation by chat
EHR: electronic health record
LUMC: Leiden University Medical Center
WMO: Medical Research Involving Human Subjects Act
